# First person – Eleanor McKay

**DOI:** 10.1242/dmm.050839

**Published:** 2024-06-28

## Abstract

First Person is a series of interviews with the first authors of a selection of papers published in Disease Models & Mechanisms, helping researchers promote themselves alongside their papers. Eleanor McKay is first author on ‘ Female Alms1-deficient mice develop echocardiographic features of adult but not infantile Alström syndrome cardiomyopathy’, published in DMM. Eleanor conducted the research described in this article while a PhD Student in Robert Semple's lab at the Centre for Cardiovascular Science, Queen's Medical Research Institute, University of Edinburgh, Edinburgh, UK. She is now a Postdoctoral Researcher in the lab of Stephan Kellenberger at University of Lausanne, Lausanne, Switzerland, investigating metabolism and related human diseases.


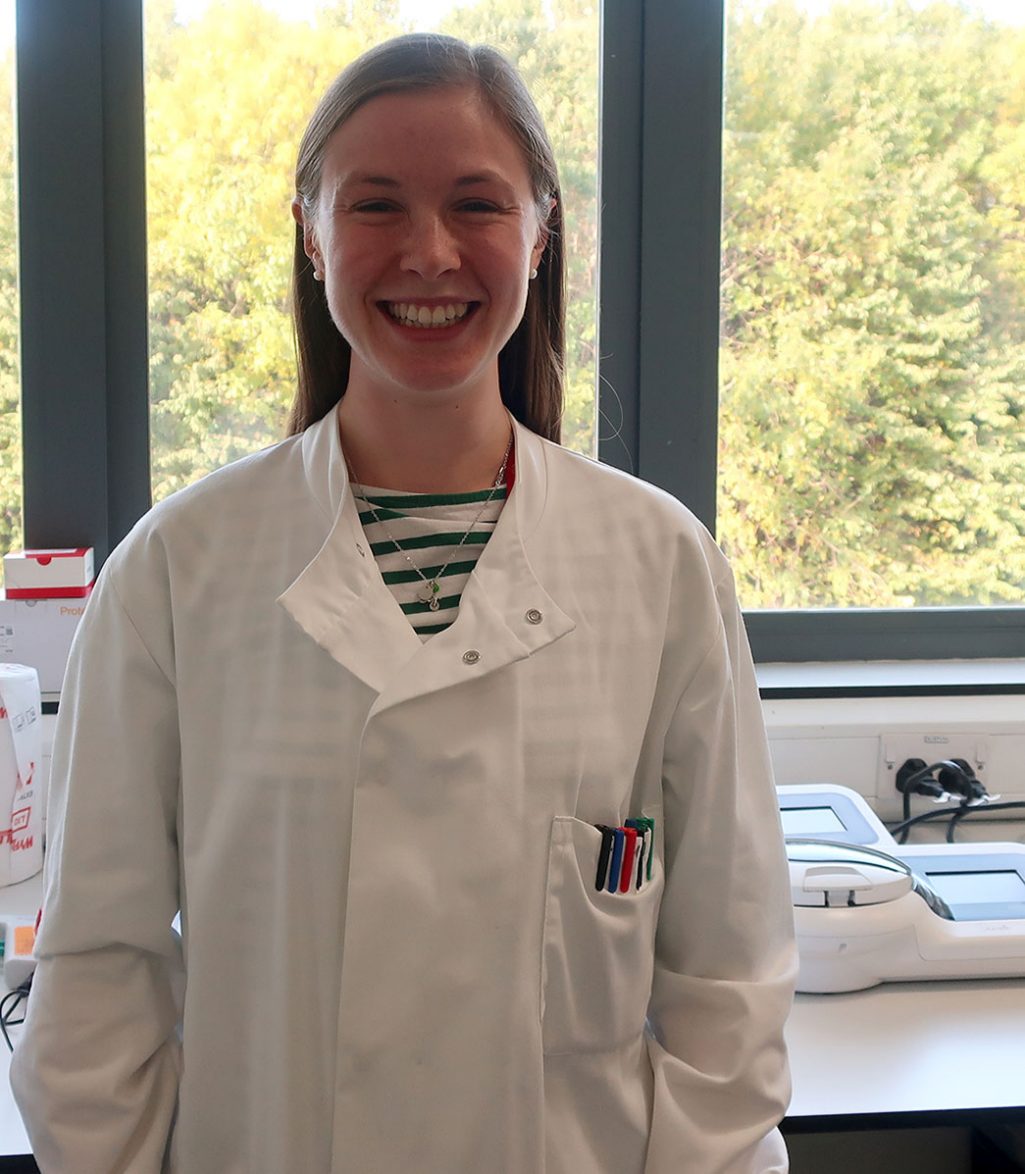


**Eleanor McKay**

**Who or what inspired you to become a scientist?**

When I was asked ‘what do you want to be when you grow up?’ I never really had a good answer. For such a long time I never realised that you could have a career in scientific research, I only heard about studying medicine and other vocational courses. But then, at school, we visited the National Institute of Medical Research in Mill Hill and got to meet a lot of the scientists there, talking to them about their research. Finally, I knew what I wanted to be when I grew up!

Since cardiac dysfunction is a leading cause of death in Alström syndrome, understanding whether this is modelled in mice is an important question

**What is the main question or challenge in disease biology you are addressing in this paper? How did you go about investigating your question or challenge?**

Alms1-deficient mice are good models to investigate many aspects of human Alström syndrome but their cardiac phenotype is largely uncharacterised. Since cardiac dysfunction is a leading cause of death in Alström syndrome, understanding whether this is modelled in mice is an important question. We used echocardiography to look at the structure and function of the heart at three different ages.

**How would you explain the main findings of your paper to non-scientific family and friends?**

Alström syndrome is a genetic disease caused by the inheritance of two copies of a ‘faulty’ gene from both parents. The gene that is faulty in Alström syndrome is called *ALMS1*, and it encodes the ALMS1 protein. People with Alström syndrome experience a wide range of health problems starting in early childhood, including vision and hearing loss, heart failure and type 2 diabetes. This paper focuses on the heart failure in Alström syndrome, which is a leading cause of death in patients. I used mice with faults in *Alms1* to study whether the heart problems seen in people with Alström syndrome is also seen in mice. If the heart problems seen in patients are mimicked by the mice with faults in *Alms1* then it might help us understand the mechanism of the heart failure and test potential treatments. I performed ultrasound analysis of mice hearts, similar to that performed in patients, to look at heart structure and function. This was performed in both infant and adult mice. Unlike in humans, no evidence of change in heart function in infant mice was found. In adults, I saw evidence of heart failure in female but not male mice. By comprehensively measuring the heart function at three time points in mice with faulty *Alms1*, I hope future work can develop the mouse model further by adding drugs that may unmask heart failure in infant and adult male mice. This will allow the mechanism of heart failure in Alström syndrome to be better understood and treated.

Our results show that many of the aspects of restrictive cardiomyopathy seen in patients is seen in adult female but not male mice

**What are the potential implications of these results for disease biology and the possible impact on patients?**

Cardiac dysfunction is a leading cause of death in Alström syndrome, so understanding whether this is modelled in mice is an important question. Our results show that many of the aspects of restrictive cardiomyopathy seen in patients is seen in adult female but not male mice. Historically, clinical studies of patients with Alström syndrome have not separated patient groups by sex, so I hope that this study will emphasise the importance of sex as a confounding factor. Additionally, I hope that the cardiac phenotypes reported in this study will provide a good baseline for future mouse studies to help understand the mechanism of cardiac dysfunction, as well as test possible treatments. During my PhD, I was lucky enough to meet some people with Alström syndrome, and they are so supportive of our research and give up a lot of their time to volunteer for research studies. I really hope that one day this research may be a piece of the puzzle to have a positive impact on the treatment of people with Alström syndrome.

**Figure:**
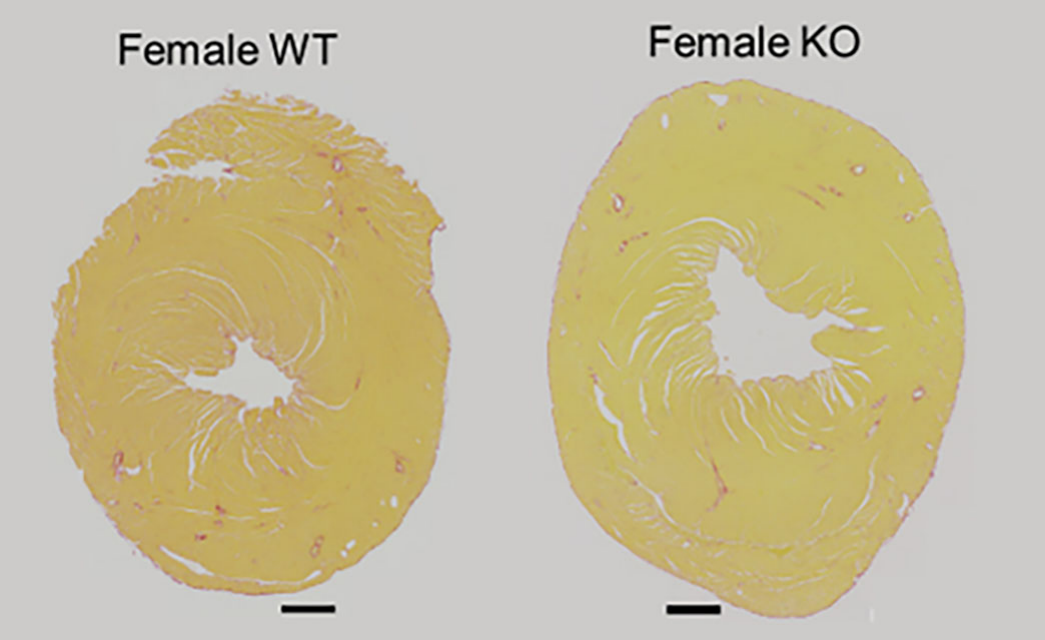
**Picrosirius-Red staining for collagen fibrosis in the heart of wild-type and *Alms1*-knockout mice.** Despite seeing a phenotype of restrictive cardiomyopathy in adult female mice lacking Alms1, we didn't see any changes in Picrosirius Red staining for fibrosis compared to WT littermate controls. Scale bars: 500 μm.

**Why did you choose DMM for your paper?**

I've been an advocate of open science since the start of my PhD, so it was fantastic to find a journal that supports many of these principles to publish my work with. I particularly like that all articles are open-access and that there is no word limit applied to the methods section. I wish more journals would abolish word limits in methods sections. I've spent too much of my life chasing down methods detailed ‘as previously described’.

**Given your current role, what challenges do you face and what changes could improve the professional lives of other scientists in this role?**

Too often I see PhD students wasting time and resources by trying to get a routine lab technique up and running, because there is nobody available to train them. The current model of academia where we promote successful lab scientists to positions where they are office based, writing grants and papers all day long, means that a lot of lab technique knowledge is frequently lost. We need more staff/senior scientists in research institutes, and we need more funding for those positions to be created. Yes staff/senior scientists are more expensive than PhD students but the experience and lab continuity that staff/senior scientists can bring is more than worth it. The swarms of PhD students spending months trying to get a simple PCR to work will be grateful too!

**What's next for you?**

I've just started a postdoc at the University of Lausanne, where I am working on the role of the acid-sensing ion channel Asic1a in metabolism and thermoregulation.

**Tell us something interesting about yourself that wouldn't be on your CV**

I love embroidery and knitting, and I like to take them to conferences and embroider or knit as I listen – it helps me concentrate! I'm currently knitting myself a stripey jumper.
